# Roles for androgens in mediating the sex differences of neuroendocrine and behavioral stress responses

**DOI:** 10.1186/s13293-020-00319-2

**Published:** 2020-07-29

**Authors:** Damian G. Zuloaga, Ashley L. Heck, Rose M. De Guzman, Robert J. Handa

**Affiliations:** 1 Department of Psychology, University at Albany, Albany, NY USA; 2 Department of Biomedical Sciences, Colorado State University, Fort Collins, CO USA

**Keywords:** Testosterone, Estrogen, HPA axis, Anxiety, Depression, Glucocorticoids, Androgen receptor, Estrogen receptor, Hypothalamus

## Abstract

Estradiol and testosterone are powerful steroid hormones that impact brain function in numerous ways. During development, these hormones can act to program the adult brain in a male or female direction. During adulthood, gonadal steroid hormones can activate or inhibit brain regions to modulate adult functions. Sex differences in behavioral and neuroendocrine (i.e., hypothalamic pituitary adrenal (HPA) axis) responses to stress arise as a result of these organizational and activational actions. The sex differences that are present in the HPA and behavioral responses to stress are particularly important considering their role in maintaining homeostasis. Furthermore, dysregulation of these systems can underlie the sex biases in risk for complex, stress-related diseases that are found in humans. Although many studies have explored the role of estrogen and estrogen receptors in mediating sex differences in stress-related behaviors and HPA function, much less consideration has been given to the role of androgens. While circulating androgens can act by binding and activating androgen receptors, they can also act by metabolism to estrogenic molecules to impact estrogen signaling in the brain and periphery. This review focuses on androgens as an important hormone for modulating the HPA axis and behaviors throughout life and for setting up sex differences in key stress regulatory systems that could impact risk for disease in adulthood. In particular, impacts of androgens on neuropeptide systems known to play key roles in HPA and behavioral responses to stress (corticotropin-releasing factor, vasopressin, and oxytocin) are discussed. A greater knowledge of androgen action in the brain is key to understanding the neurobiology of stress in both sexes.

## Introduction

The reproductive hormones, estradiol (E2) and testosterone (T), act on the brain to control important neurobiological functions, which include stress-related behaviors, cognition, neuroendocrine function, autonomic function, feeding, and metabolism [1–4]. The neurobiological effects of E2 and T are partly linked to their regulation of hypothalamic function, particularly the hypothalamic-pituitary-adrenal (HPA) axis, through changes in circulating glucocorticoid (GC) levels. Not only can GCs affect behaviors, but chronic elevations of GCs promote the development of neurological disorders [5], cardiovascular disease [6], obesity, and metabolic syndrome [7]. Clinical and preclinical studies show compelling evidence for a link between anxiety and depression, cardiometabolic disease, and obesity, with a dysregulation of the HPA axis [8, 9]. Moreover, sex differences are present in the risk for such disorders that may be related to gonadal hormone actions, as both E2 and T modulate the HPA axis [10]. Although much is known about the cellular and molecular actions of estrogens and how these hormones can regulate the HPA axis and stress-related behaviors, much less has been published regarding role(s) for androgens in stress hormone and behavioral responses to stress. In this review, we will address the multimodal actions of androgens in modulating the HPA axis as well as its neurobiological actions that can regulate stress-related behaviors. These include not only androgens acting through classical androgen receptor (AR) mechanisms, but also androgen metabolism to compounds that may bind with differing affinities to the AR or act through binding to estrogen receptors (ERs).

## Overview of the hypothalamic-pituitary-adrenal axis

Animals respond to threats to their welfare with the activation of neurons that control the HPA axis, behavioral responses, and autonomic responses appropriate for the context into which they are placed [11]. The HPA axis, in particular, is a stressor-activated network that facilitates essential, body-wide adaptations to challenges by connecting hypothalamic neurons with the pituitary and, in turn, adrenal glands, which secrete stress hormones to maintain homeostasis. For HPA axis activation, the paraventricular nucleus of the hypothalamus (PVN) is a critical regulatory node and is the final common pathway for the stress hormone responses. The PVN integrates multiple inputs from various upstream limbic regions which access the PVN trans-synaptically through neurons in the bed nucleus of the stria terminalis (BST) and peri-PVN region (see [3] for review). For the HPA axis, the PVN’s output is ultimately coordinated by a group of neurons that synthesize a 41 amino acid peptide, corticotropin-releasing factor (CRF). CRF-expressing neurons send projections to the external zone of the median eminence where they can secrete CRF into the hypophyseal portal vasculature, thereby connecting the hypothalamus with cells in the anterior pituitary gland. CRF can then act upon corticotropin-releasing factor receptor 1 (CRFR1), a G-protein-coupled receptor found on corticotrophs, to allow the secretion of ACTH from the anterior pituitary gland into the general circulation. ACTH subsequently drives the adrenal secretion of GCs. Cortisol, the predominant GC in humans, and corticosterone (CORT), the predominant GC in rats and mice, can then act upon peripheral tissues to mobilize energy stores, induce lipolysis and proteolysis, and potentiate vasoconstriction driven by the autonomic nervous system (ANS), among myriad other effects. In addition, GCs have important actions within the central nervous system. Chronically elevated GCs can suppress reproduction and immune function and alter behaviors [11, 12]. Importantly, acute elevations in GCs that occur following stressors are thought to be beneficial and enhance cognition and metabolism and inhibit inflammatory responses. Together with the responses of the autonomic nervous system, these become detrimental with excess or chronic activation of the HPA axis. Chronic elevations in GCs have deleterious effects on neural function by decreasing the resilience of neurons and glia, thereby promoting neurotoxicity [13, 14]. Such elevations are also associated with obesity, insulin resistance, and neurological disorders [5, 7]. Thus, understanding the factors regulating the HPA axis is important for ultimately controlling GC excess and disease risk.

## Sex differences in the HPA axis in rodents and humans

**Fig. 1:**
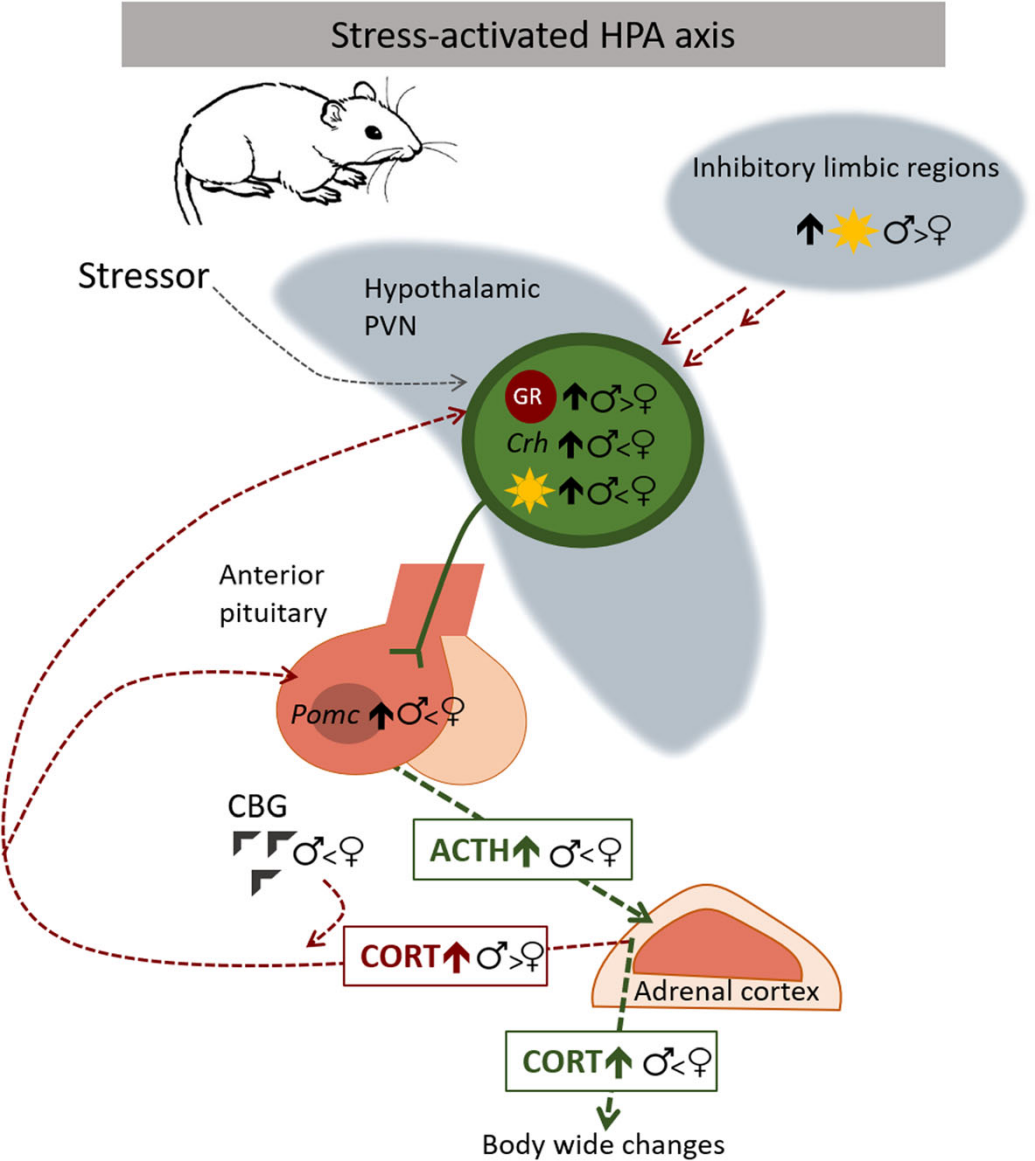
Sex differences in HPA axis response to stress. Adult male versus female rodents have decreased HPA axis responses to acute stressors characterized by decreased paraventricular nucleus (PVN) neuronal activation and corticotropin-releasing hormone (CRH) gene expression, decreased pituitary expression of the proopiomelanocortin (POMC) precursor for adrenocorticotropin (ACTH), and decreased ACTH and corticosterone (CORT) responses to acute stressors. Males also have enhanced negative feedback (red arrow) resulting from their reduced corticosteroid-binding globulin (CBG) levels, their increased PVN glucocorticoid receptor (GR) gene expression, and their increased neuronal activation in limbic regions that inhibit the HPA axis. The sun symbol indicates neuronal activation

Sex differences occur in the rodent’s HPA axis response to stress, with females having a higher baseline level of CORT and a more robust CORT and ACTH response after exposure to a number of different types of acute stressors [15–18] (Fig. 1). Correspondingly, females have greater stress-responsive neuronal activity [18–20] and CRF gene expression in the PVN, as well as mRNA levels of the ACTH precursor proopiomelanocortin (POMC) in the anterior pituitary [15, 17–19]. Additionally, female rats have a delayed return to baseline levels of ACTH and CORT after acute stress, suggesting sex differences in the negative feedback regulation of the HPA axis [15–18]. In limbic structures known to activate inhibitory inputs to the HPA axis, such as the frontal cortex and hippocampus, females have reduced neuronal activity compared to males following acute restraint [21]. Sex differences also exist in glucocorticoid feedback mechanisms that ultimately contribute to the less robust negative feedback on the HPA axis in females. Female rats have been shown to have lower hypothalamic glucocorticoid binding [22], reduced density of corticosteroid receptors in the pituitary [23], and lesser upregulation of hypothalamic corticosteroid receptor mRNA after acute stressors than do males [24]. Moreover, loss of forebrain [25] or PVN [26] GR results in HPA axis dysregulation in male but not female mice, suggesting that GR is less important for HPA regulation in females. Although the females used in these studies were not characterized according to stage of the estrous cycle, the data are consistent with that of Heck et al. [27] who later showed that negative feedback by glucocorticoid receptors is lacking in females in proestrus when estrogen levels are highest.

The availability of corticosteroids in brain regions where they can modulate HPA axis function may also influence their sex-dependent actions. While in the circulation, most corticosteroids are bound by corticosteroid-binding globulin (CBG), a liver glycoprotein [28]. At the target, CBG releases corticosteroids (see [29] for review), since they can only bind the GR or MR when free from CBG [30]. Therefore, CBG levels can directly influence the activity of CORT by adjusting the available hormone to act upon target tissues. In females, basal CBG levels are almost twice those in males, yet levels of free CORT are not statistically different [31]. This raises the possibility that the higher levels of CBG may be buffering the higher total CORT in females [31, 32]. However, it is also possible that higher female CBG levels make them less responsive to negative feedback on the HPA axis following acute stress, as corticosteroids can only bind their receptors when free from CBG. In males, CBG is negatively regulated by acute stress, which makes CORT more available for negative feedback [33]. It is unknown if this is true in females. Thus, elevated CBG levels in females could make CORT less available for negative feedback and contribute to the enhanced HPA axis response to stress often reported in female rodents.

Notably, sex differences in the activity and regulation of the rodent HPA axis can depend on numerous factors, including strain and housing condition [34–39]. Nonetheless, most studies report that male rodents show a blunted HPA axis response to acute stress relative to females. In contrast, sex differences in the HPA axis are much less consistent in humans [40, 41]. While studies suggest that women have enhanced HPA responses to acute stressors compared to males, others indicate greater responses in males or no significant difference [40, 42–45]. These contradicting findings could be due to a number of factors, with stressor modality, age, health, and menstrual cycle stage of the participants being of importance [40, 42, 44].

## Sex differences in anxiety/depressive-like behaviors in humans and in rodents

Dysregulation of the HPA axis (hypo- or hyper-reactivity) is a hallmark of many stress-related diseases, which are known to differentially present in men versus women [40]. For instance, women are twice as likely to be diagnosed with depression and anxiety than are men [46–48], and subclinical symptoms of depression and anxiety are also more frequently reported in women [46–50]. Sex-related differences in HPA axis activity that may contribute to this sex difference are the focus of this review. However, the autonomic nervous system has also been implicated in mood disorders, and sex differences within its activity are an important area of research that requires further investigation [51].

Unlike in humans, sex differences in anxiety- and depressive-like behavior are inconsistently reported in rodents. Several studies in rats have reported that female rats show decreased anxiety-like behaviors, particularly in the elevated plus maze, compared to males [52–54]. In assays of depression-like behaviors, such as the forced swim test and sucrose preference test, female rats have also been demonstrated to show decreased despair-like behavior compared to males [55, 56]. However, many other studies reported no sex differences in these behaviors (e.g., [57]) and some have reported decreased anxiety- and depressive-like behaviors in male rats [58]. In mice, sex differences in anxiety- and depressive-like behaviors are inconsistently reported [59, 60], and also similar to rats, the appearance of sex differences varies by genetic background as reflected by strain differences [59, 61, 62]. Sprague Dawley rats, for example, exhibit stable sex differences in immobility measures in a modified and extended version of the forced swim test, whereas a lack of sex differences was present in Long Evans rats across tests [63]. Similarly, housing environment can influence sex differences in affective behaviors in rodents [64, 65].

It is important to note however that behavioral assays for detecting anxiety or depressive-like behaviors in rodents were originally designed to screen potential anxiolytic and antidepressant compounds for activity and not for describing such behaviors in untreated animals. Thus, sex differences in such behavioral approaches may not provide an accurate assessment of basal mood state. It is also important to note that male and female rodents are reported to show differences in motivations underlying behaviors in anxiety-related tests such as the elevated plus maze. Factor analysis studies indicate that elevated plus maze behavior in male mice and rats is primarily motivated by anxiety, whereas behavior in females is primarily motivated by activity [66, 67]. These studies suggest that interpretations of behaviors in anxiety-like assays might differ between males and females. Anxiety-like behaviors can also vary depending on estrous cycle phase in female rats, which is another important factor to consider when assessing sex differences in rodents [68].

The studies in rodents discussed above primarily involved assaying sex differences in anxiety- and depressive-like behaviors in rodents that had not been pre-exposed to prior stress. Importantly, select chronic stress models have been demonstrated to induce sex-specific effects on anxiety- and depressive-like behaviors, as well as HPA axis responses in mice. One such model is a sub-chronic variable stress (CVS) paradigm that involves exposure to varying stressors over a 6-day period [69]. This CVS model increases anhedonia-like behaviors in the sucrose preference test, increases anxiety-like behaviors in the novelty suppressed feeding task, and elevates CORT levels in female but not male mice [69, 70]. In contrast, other chronic stress paradigms, which are generally longer in duration and include greater stress severity, have yielded mixed results including greater behavioral disturbances in males or increased anxiety/depressive-like behaviors in both sexes [71–73]. Collectively, these studies suggest that sub-chronic variable stress can induce sex differences in anxiety and depressive behaviors that are reflective of the human population (males < females), which is critical in terms of increasing translational value and for understanding mechanisms that might underlie female vulnerability or male resistance to stress-related disorders.

### A role for gonadal hormones

While social, cultural, and experiential factors contribute to sex differences in anxiety and depression [74, 75], it is also clear that gonadal steroid hormones are a key contributing factor [76]. Sex differences in the prevalence of anxiety and depression develop after puberty [77]. Additionally, it has been postulated that the greater prevalence of depression in women [46, 78] is related to female-specific reproductive events marked by fluctuations in gonadal hormones, such as perimenstrual changes, pregnancy, the postpartum period, and menopause (see [79, 80] for review). Behavioral changes are also associated with hysterectomy, use of oral contraceptives, and use of testosterone therapy for reduced libido in women [81–83]. Furthermore, time-dependent exposure to stressors or glucocorticoid administration during development can influence the adult physiology of one sex more than the other in humans and animals [77] [84–89], supporting a role for perinatal and adult levels of gonadal hormones to alter adult risk for stress-related diseases.

As the cellular and molecular actions of estrogens and their effects on the HPA axis and stress-related behaviors are relatively well-defined, this review will focus on the androgen influences on stress hormone and behavioral responses to stress.

## Androgens regulate the HPA axis and stress-related behaviors

### Activational actions of androgens on the HPA axis and stress-related behaviors

**Fig. 2:**
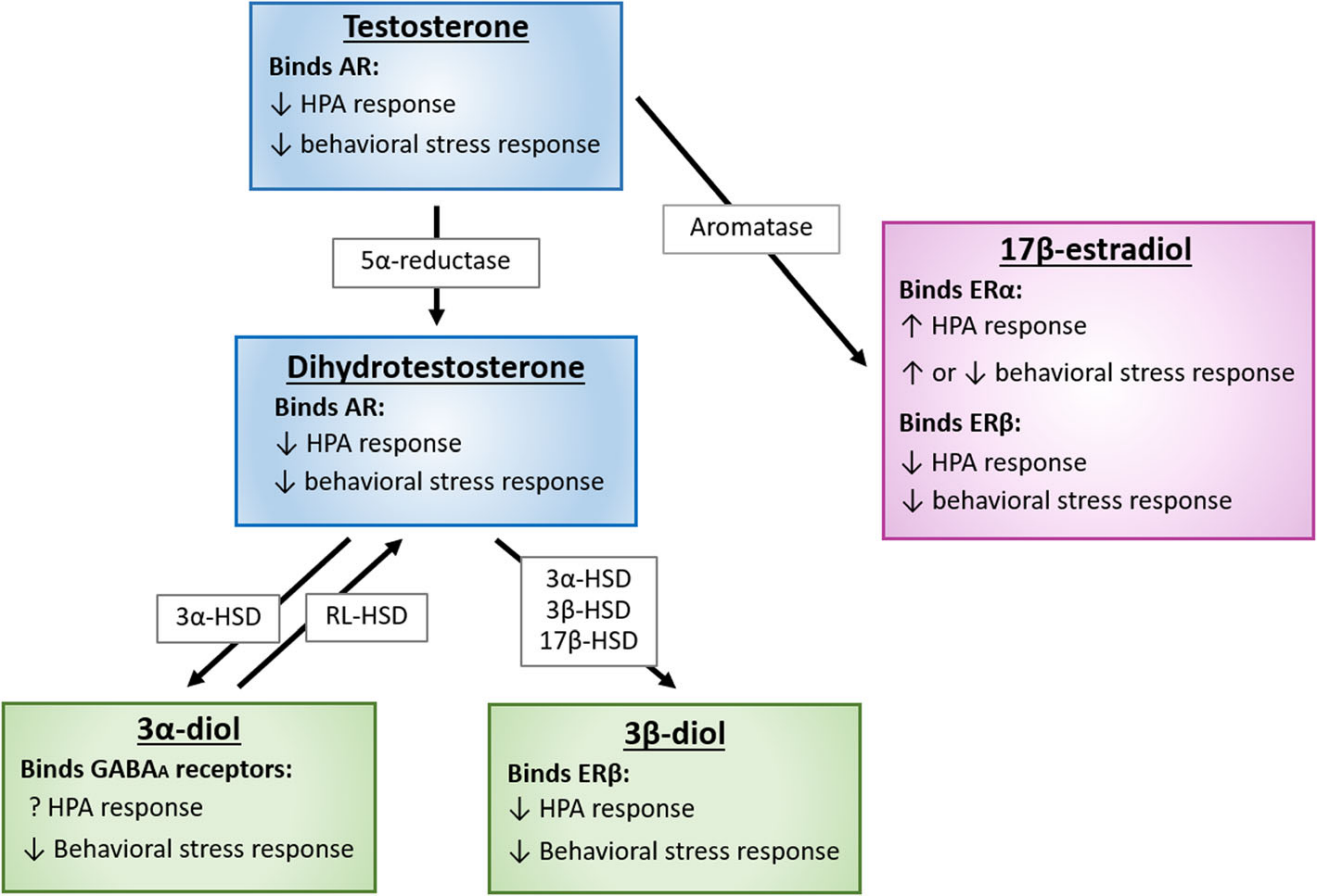
Effects of testosterone (T) and its metabolites on HPA axis and behavioral stress responses. This figure describes enzymes involved in the conversion of T and its metabolites and predicted effects produced by binding AR, ERα, ERβ, and GABA receptors. Binding of AR or ERβ is expected to decrease HPA axis and behavioral stress responses. In contrast, actions at ERα increase the HPA axis response to stress while effects on anxiety-like behaviors are mixed and depend on reproductive status among other factors. Effects of 3α-diol on the HPA axis are currently unknown. HSD = hydroxysteroid dehydrogenase; 3α-Diol = 5α androstane 3α, 17β Diol; 3β Diol = 5α androstane 3β, 17β Diol, RL-HSD = 11-cis-retinol dehydrogenase like 3α-HSD

Androgens are generally thought to inhibit the activity of the HPA axis. Thus, removal of endogenous androgens by gonadectomy (GDX) in males increases stress-induced ACTH and CORT secretion, and T treatment has the opposite effects [19, 90, 91]. T modulates the HPA axis either through direct actions at ARs or via metabolism to other compounds that interact with ARs or ERs (see Fig. 2). Notably, the reduction of T to the more potent and non-aromatizable androgen, dihydrotestosterone (DHT), is necessary for its suppression of glucocorticoid secretion after stress [92]. Accordingly, inhibition of the enzyme that converts T to DHT, 5α-reductase, with the central infusion of finasteride increases stress-enhanced glucocorticoid secretion in male rats [92]. Consistent with these results, DHT placed near the PVN in adult, gonadectomized (GDX’d) male rats reduces the ACTH and CORT responses to acute stress [93].

An important point to consider is that most studies examining androgen effects on the HPA axis employ DHT, which has a very high affinity for ARs, and cannot be aromatized to E2 [94]. However, DHT has another interesting property in that it can be metabolized to 5α-androstane-3β, 17β-diol (3β-diol), which has been shown to bind and activate estrogen receptor beta (ERβ) [94]. Like DHT and ERβ agonists, 3β-diol inhibits the HPA axis response to stress [93, 94]. These data suggest that the inhibitory effects of DHT on HPA axis activity may be partially mediated by 3β-diol signaling through ERβ and emphasize the importance of considering the roles ERβ may play in facilitating androgen regulation of the HPA axis. Another pathway through which androgens can potentially regulate the HPA axis is via conversion of T to E2 by the aromatase enzyme. E2 potently binds both estrogen receptor alpha (ERα) and ERβ and can modulate HPA function through actions at both receptors.

Androgens have also been repeatedly shown to reduce anxiety and depression symptomology. Anxiety and depression are elevated in hypogonadal men and aging men with low levels of T [95–98]. Androgen replacement in older men and hypogonadal men elevates mood and decreases depressive symptoms, indicating that low levels of androgens specifically contribute to the etiology of these disorders [98, 99]. Furthermore, T has been shown to attenuate cortisol release in men [100]. The androgenic suppression of hypothalamic-pituitary-adrenal (HPA) axis responses may play a key role in decreasing susceptibility to develop anxiety and depression, since chronic high levels of glucocorticoids are a key contributor to the development of these disorders [101].

In rodent models, androgens and their metabolites have also been shown to modulate anxiety- and depressive-like behaviors [3, 102]. Male rats GDX’d as adults show increased anxiety- and depressive-like behaviors, and these effects are eliminated by supplementation with T [103–105]. Similar androgen effects on anxiety- and depressive-like behaviors have been reported in mice [70, 106–109]. Furthermore, procedures that increase endogenous T (e.g., exposure to females and female urine) also decrease anxiety-like behaviors [110, 111]. Androgens have been shown to produce anxiolytic effects via metabolism of T to several different compounds including, but not limited to DHT, androsterone, E2, 3α-diol, and 3β-diol. Treatments with 3α-diol and E2 have both been demonstrated to produce anxiolytic effects in male mice in the open field and light-dark box test and anti-depressant effects in the forced swim test [112, 113]. Furthermore, androsterone, DHT, and 3β-diol treatments have all been shown to reduce anxiety-like behavior in a variety of tests [111–114]. These effects of T and its metabolites on both the HPA axis and behavioral stress responses are proposed to be mediated through ARs, ERs, and GABA receptors [94, 111].

### Organizational actions of androgens on the HPA axis and stress-related behaviors

#### Perinatal hormones

Development of sex differences in rodents has largely been attributed to perinatal exposure to T that occurs only in males, particularly a prenatal surge in late gestation and a second surge that occurs immediately after birth [115, 116]. These androgen pulses serve to either increase or decrease specific phenotypes of neurons in males [116, 117]. Typically, these effects occur following conversion of T to E2 by the aromatase enzyme and subsequent binding to ERα [115]. Humans, like rodents, are exposed to a gestational T surge which is proposed to regulate analogous sex differences in the human brain [117]. This differential exposure to gonadal hormones during development and adulthood alters brain structure and function and ultimately contributes to sexual dimorphisms in behavior and hormone release [117].

Gonadal hormone exposure around the time of birth has been shown to organize HPA axis functions in rodents [117]. Neonatal GDX in males increases basal and stress-induced CORT responses to stress in adult rats [118, 119]. Additionally, a single injection of T in females on the day of birth decreases their adult HPA axis responses to stress [120] and reduces the amplitude and frequency of the pulsatile pattern of CORT secretion that is characteristic of female rats [120]. On the other hand, inhibition of the aromatase enzyme during the neonatal period in male rats increases stress-induced CORT and neural activation in stress-associated brain areas in adulthood [121]. This suggests that neonatal T, likely via metabolism to E2 and interactions with ERs, suppresses adult stress reactivity.

Perinatal gonadal androgens have also been demonstrated to modulate adult anxiety-like behaviors although effects of androgens are commonly reported to be anxiogenic as opposed to generally anxiolytic effects found following adult treatments. Treatment of pregnant rats with T from gestational day 15 to 19 increased anxiety-related behaviors in offspring in the elevated plus maze, and this effect was strongest in females [122]. Furthermore, neonatal T treatment in female mice increases anxiety-like behaviors in the marble burying task, although it did not affect depressive-like behaviors in the tail suspension test [109]. Similarly, neonatally GDX’d male rats show decreased anxiety-like behaviors in several assays including the open field test, light-dark box, and elevated plus maze compared to mice that remained gonadally intact during the neonatal period [118, 123].

#### Pubertal hormones

Increases in circulating gonadal steroids occur at puberty and can provide another organizing signal to drive permanent changes in brain function. Accordingly, some evidence suggests that puberty represents a second critical period during which organizational actions of gonadal hormones can further influence the HPA axis [124]. Pubertal gonadal hormones modulate the neural and behavioral changes that ultimately produce adult-like characteristics [124], including changes in stress hormone responses. Prepubertal rats of both sexes have greater glucocorticoid responses to acute stressors compared to their responses in adulthood ([125, 126] for review see [127]). T exposure during both the prepubertal and perinatal period is important for the masculinization of HPA responses to stress in adulthood [109]. Pubertal T exposure is thought to organize the adult male-typical sensitivity of the HPA axis to androgen regulation [128]. Unlike males GDX’d in adulthood, adult male rats that were GDX’d before puberty do not respond to T administration with decreases in basal or stress-induced PVN gene expression or glucocorticoid secretion [128]. Thus, puberty represents a potential critical organizational period during which rising levels of androgens play an important role in sculpting the correct development of a male-typical, androgen-sensitive adult HPA phenotype (for review, see [102]).

Pubertal elevations in E2 may also play an important, although less pronounced, role in the organization of the adult HPA axis. Before puberty, E2 decreases HPA axis activity in female rats, whereas in adulthood it increases HPA activity [129]. Interestingly, this change in the HPA response to E2 does not appear to depend on prepubertal elevations in ovarian steroids [129]. In adult females, E2 treatment increases basal and stress-induced CORT secretion regardless of whether they were ovariectomized before or after puberty [129]. Thus, unlike that for androgens, pubertal E2 exposure is not essential for establishing the stimulatory effects of E2 on the HPA axis in adult females.

Gonadal hormones during puberty also regulate stress-related behaviors, although effects appear to be species specific. In rats, GDX in males prior to or near the onset of puberty has been shown to decrease anxiety-related behaviors in the open field, elevated plus maze, and light-dark box [130, 131]. These results are similar to those found in rats GDX’d in the neonatal period and raise the possibility that puberty rather than the neonatal period might be the critical phase for gonadal hormone organization of anxiety-like behaviors; however, further experiments that involve hormone manipulations at both periods are necessary to assess this possibility [118, 123]. In contrast to rats, pre-pubertal GDX in male mice increases, while pre-pubertal GDX in females decreases, anxiety-like behaviors [132, 133]. Together, these studies suggest that pubertal gonadal hormones contribute to the organization of anxiety-like behaviors, although the direction of effects differs by species in rodents.

## Gonadal steroid hormone receptors involved in androgen effects on the HPA axis and anxiety/depressive behaviors

### Androgen receptor

#### HPA axis

As mentioned above, T and DHT have been well established as potent suppressors of the HPA axis [16, 90]. Furthermore, antagonism of AR with flutamide or finasteride increases CORT responses to stress [92, 134]. However, both T and DHT can be further converted to compounds that bind estrogen, GABA, and other receptors [94, 135, 136]. Therefore, studies performed in AR-deficient rodents in the past decade have been vital in confirming a critical role of AR in suppressing HPA axis reactivity [106, 108, 137, 138]. In a series of studies performed in mice and rats with the spontaneously occurring testicular feminization mutation (Tfm), which renders the AR largely non-functional, Tfm males demonstrated increased CORT responses to stress compared to wild types [106, 138]. It is important to note that the Tfm rat has elevated T levels compared to wild-type mice [139], yet still shows an elevated CORT response to stress, providing further evidence that AR is necessary for androgen suppression of the HPA axis. The role of AR in HPA axis regulation has been further supported in experiments utilizing a Cre-lox AR knockout mouse line (iTfm). Both basal and stress-induced (light-dark box) CORT levels are elevated in T-treated iTfm mice compared to similarly treated wild-type mice [108].

#### Anxiety/depressive-like behaviors

Actions of T and its metabolite DHT have been shown to produce anxiolytic effects via actions at the AR. Treatment with flutamide reduces the anxiolytic effects T in orchidectomized rats [114]. Studies involving various lines of AR-deficient mice and rats further support the role of AR in influencing anxiety- and depressive-like behaviors. Both mice and rats with the Tfm mutation show increased anxiety-like behaviors in several assays [106, 138, 140]. Similar findings of increased anxiety-like behavior are reported in the iTfm model [108]. A recent study also indicates that the male resilience to exhibit anhedonia-like behavior following sub-chronic variable stress is regulated by T [70]. Furthermore, depressive-like symptomology is enhanced in AR-deficient mice exposed to chronic mild stress [141]. Together, these studies indicate a robust role of androgen acting via AR in influencing anxiety- and depressive-like behaviors in rodents. Studies in humans also indicate that androgen actions through the AR are beneficial to mood, since treatment with flutamide in prostate cancer patients increases depressive symptoms [142, 143]. Furthermore, individuals with complete androgen insensitivity syndrome, who have dysfunctional AR, are reported to show high rates of depression (~ 36% incidence [144];) and AR is decreased in the PVN of depressed patients [145].

#### Anatomical sites of action for AR effects on the HPA axis and stress-related behaviors

Androgens have been suggested to act via AR at the levels of the pituitary and adrenal glands to modulate the HPA axis. AR knockout mice show increased POMC mRNA and decreased GR in the pituitary as well as hypertrophic adrenal glands [146]. However, the possibility that AR knockout results in changes at the PVN level, which in turn lead to this increased POMC mRNA and adrenal hypertrophy, cannot be excluded. Accordingly, there is strong evidence that T acts via AR centrally within the brain to regulate the HPA axis, since inhibitory effects of androgens on ACTH and CORT release are blocked by administration of the 5-alpha reductase inhibitor, finasteride into the 3rd ventricle [92].

Several brain regions have been implicated as potential sites at which androgens might interact with AR to regulate the HPA axis and anxiety- and depressive-like behaviors. A study comparing expression of the immediate early gene c-Fos in the brains of Tfm and wild-type rats subjected to an open field stress revealed differing neural activation patterns in the dentate gyrus, medial preoptic area, and medial amygdala, suggesting these as sites at which actions at AR might mediate stress functions [138]. However, more evidence indicates the BST or PVN as sites for such actions. The BST contains very high levels of AR within nearly all subdivisions in rodents [147, 148]. AR-containing cells in the BST project to the parvocellular PVN and thus have been proposed as a site at which androgens can regulate HPA axis reactivity [149, 150]. The BST is also well known to play a key role in influencing anxiety-related behaviors and has been suggested as a critical brain site for mediating sex differences in anxiety and depression [151]. The PVN, on the other hand, contains moderate levels of AR. While few CRH-containing neurons co-express AR [27, 152], there is evidence that AR acts outside these neurons, but still within the PVN, to influence HPA activity [27, 153–155]. Additionally, although the PVN has historically been associated with regulating neuroendocrine and autonomic functions, it has become clear that this region also controls behavioral responses to stress [156]. Thus, the PVN, like the BST, is a likely site at which actions at AR influence the HPA axis and stress-related behaviors.

### Estrogen receptor alpha

#### HPA axis

Another potential mechanism for androgen regulation of the HPA axis is through conversion of T to E2 and subsequent binding to ERα. In contrast to the inhibitory role of AR, binding to ERα activates the HPA axis. Stimulation of ERα using the selective agonists propylpyrazoletriol (PPT) and moxestral increases stress-induced ACTH and CORT in male and female rats [93, 157]. ERα has also been shown to play a key role in diminishing negative feedback on the HPA axis. Administration of PPT impairs the ability of dexamethasone to suppress the diurnal rise and restraint stress-induced release of ACTH and CORT [158]. It is important to note that while androgens are capable of producing these effects via further metabolism and binding of ERα, systemic T generally suppresses stress-induced HPA axis responses and increases negative feedback on the HPA axis [16, 159]. This suggests that interactions with other gonadal steroid hormone receptors might negate or diminish any HPA axis enhancing effects of ERα.

#### Anxiety/depressive-like behaviors

Actions through ERα have commonly been shown to produce anxiogenic effects in rodents. This anxiety-enhancing role of ERα is supported by studies involving pharmacological stimulation of ERα with PPT, which has been shown to increase anxiety-like behaviors [157, 160]. However, the role of ERα remains somewhat unclear [160–162], as both anxiogenic and anxiolytic effects of PPT that depend on the reproductive experience of females [163, 164] have been reported, and few investigations of ERα’s role in stress responsivity have been performed in males [160]. Interestingly, discrepant effects of global ERα deletion have been reported in male and female mice. For example, deletion of ERα fails to alter anxiety-like behaviors in females [165] but increases anxiety-like behaviors in males [166]. This indicates that in males, ERα binding might normally reduce anxiety-like behaviors. However, this interpretation is complicated, and both organizational and activational roles of ERα in influencing these behaviors need to be considered since ERα is absent in ERα knockout mice throughout the lifespan. In human populations, specific variants of the ERα gene have been repeatedly associated with anxiety and depression disorders [167–169]. This indicates that ERα might also be a key receptor through which sex steroid hormones can affect anxiety/mood disorders in humans.

#### Anatomical sites of action for ERα effects on the HPA axis and stress-related behaviors

Given that there are few ERα expressing cells in the rat PVN [170], and only modest levels in the mouse PVN [171], one key site implicated in regulating the HPA axis enhancing effects of ERα is the peri-PVN. The peri-PVN is located just dorsal and lateral to the PVN and contains high levels of ERα [172, 173]. Infusion of PPT into the peri-PVN in rats suppresses dexamethasone-induced negative feedback on the HPA axis and increases psychological stress-induced c-Fos within the PVN [158]. These findings indicate that peri-PVN ERα is critical for central regulation of HPA axis function. The peri-PVN is primarily comprised of GABAergic neurons, thus actions at peri-PVN ERα are predicted to decrease inhibitory tone to PVN HPA controlling neurons, thus increasing HPA axis activation [3].

In general, the neuroanatomical sites within which ERα can induce effects on stress-related behaviors are largely obscure, although the nucleus accumbens, preoptic area, hypothalamus, and amygdala have been suggested [3, 174–176]. Furthermore, ERα has been shown to have opposing effects, both increasing and decreasing anxiety- and depressive-like behaviors depending on the brain region in which it is located. Downregulation of ERα in the medial preoptic area and posterodorsal amygdala decreased anxiety-like behavior suggesting an anxiogenic role of ERα in these regions [175, 176]. In contrast, ERα overexpression in the nucleus accumbens induces anti-depressant-like effects and stress resilience in male and female mice pre-exposed to chronic stress [174]. These region-specific, opposing actions on stress-related behaviors may contribute to discrepant findings involving systemic PPT administration and global deletion of ERα [160, 165, 166].

### Estrogen receptor beta

#### HPA axis

Androgens can interact with ERβ via conversion of T to E2 by the aromatase enzyme. Furthermore, the 5-alpha-reduced T metabolite DHT can be further converted to 3β-diol, which has a high affinity for ERβ [177], but very low affinity for AR [178]. Numerous studies in rodents indicate an HPA axis-suppressing role of ERβ. Administration of the ERβ agonist DPN attenuates stress-induced increases in ACTH and CORT in male and female mice and rats [93, 157, 158, 179], while DPN produces no effects in ERβ knockout mice [179]. Studies in GDX’d males also indicate that administration of the androgen metabolite 3β-diol attenuates stress-induced ACTH and CORT [93, 180]. Furthermore, co-administration of the ER antagonist tamoxifen to DHT-treated mice partially blocks the inhibitory effects of DHT on ACTH and CORT responses [93]. It is proposed that this partial inhibition occurs via blockade of 3β-diol actions at ERβ [3, 93].

#### Anxiety/depressive-like behaviors

In contrast to ERα, actions at ERβ have primarily been shown to induce anxiolytic effects. ERβ knockout female mice show increased anxiety-like behaviors, although these behaviors were not altered in ERβ knockout males [165]. Furthermore, administration of the ERβ agonists DPN and WAY-200070 reduces anxiety-like behavior in male and female rodents in a variety of tests including the open field test and elevated plus maze [157, 158, 179]. Again, the likely mechanism through which androgens might decrease anxiety-like behaviors via actions at ERβ is through conversion of DHT to 3β-diol and subsequent binding of ERβ. Administration of 3β-diol has been shown to decrease anxiety-like behavior in the elevated plus maze [181], and this effect is dependent on the presence of functional ERβ, since 3β-diol fails to produce an anxiolytic effect in ERβ knockout mice [182]. Fewer studies have investigated a role of ERβ in influencing depressive-like behaviors; however, a recent report suggests a sex-specific role of ERβ in regulating stress-induced anhedonia. Male ERβ knockout mice show decreased sucrose preference in response to inescapable footshock, while preference in females was unaffected [183]. Evidence in humans for a potential role of ERβ in stress-related behaviors is quite limited in comparison with rodent literature. Two studies reported that AA alleles of Esr2 rs1256049 and rs4986938 were associated with an increased incidence of generalized anxiety disorder and major depression, respectively, in women [168, 184]. However, the role of Esr2 variants in men, and the contribution of the role of androgens, is poorly understood.

#### Anatomical sites of action for ERβ effects on the HPA axis and stress-related behaviors

One potential key site for ERβ modulation of the HPA axis is the PVN, which expresses very high levels of ERβ in both rats and mice [171, 173, 185, 186]. Implantation of DPN near the PVN in male rats attenuates stress-induced ACTH and CORT and c-Fos expression in the PVN [93], similar to peripheral injections of DPN [157]. Importantly, the PVN contains the key enzymes needed for metabolism of androgens to ER ligands including aromatase, 5α-reductase, 17β-HSD, 3α-HSD, and cytochrome p450 7b (CYP7B) [93, 187]. ERβ may also directly regulate HPA axis activation, as neuropeptide-expressing PVN neurons known to modulate HPA activity co-express ERβ [171, 188] [171, 185, 188, 189]. Alternatively, the BST also contains high levels of ERβ and might be a key site for ERβ regulation of the HPA axis based on its known role in modulating HPA axis tone [3, 186].

One ERβ-rich region, the dorsal raphe nucleus, has also been proposed as a potential key site for influencing stress-related behaviors [190]. Dorsal raphe serotonin neurons exhibit high co-expression with ERβ in rats [191] and non-human primates [192]. There is strong evidence that estrogen actions through ERβ can regulate the serotonin system by modulating levels of tryptophan hydroxylase, the enzyme required for serotonin synthesis. Ovariectomy decreases, while supplementation with the ERβ agonist LY3201 increases, dorsal raphe tryptophan hydroxylase [193]. Placement of DPN near the dorsal raphe can increase TPH2 mRNA expression and also increase active stress-coping behaviors in the forced swim test [191]. Furthermore, tryptophan hydroxylase is decreased in the brains of ERβ knockout male and female mice [193]. Castration in males has also been shown to decrease dorsal raphe serotonin synthesis while supplementation with T increases synthesis [194, 195]. Therefore, androgen metabolites might also induce effects on dorsal raphe serotonin neurons by binding ERβ, although this has not been directly tested. A recent study revealed that injections of DPN into the dorsal raphe reduced anxiety-like behaviors in food-restricted female rats, further implicating this as a key site of action for influencing behavioral stress-responses in rodents [196]. However, in non-food-restricted female rats, dorsal raphe administration of DPN failed to alter anxiety-like behaviors [191]. The potential role of androgens in this process is largely unclear, in part due to the absence of similar studies performed in males. Alternative sites for ERβ’s effects on stress-related behaviors are the PVN, BST, and medial amygdala given the high expression of ERβ and known role of these regions in influencing behavioral stress responses [156, 165, 172, 179, 186].

### Gonadal hormone receptor signaling

AR, ERα, and ERβ all belong to the same family of receptors, nuclear receptor subfamily 3, and all receptors in this subfamily function classically as ligand-activated transcription factors [197]. They reside as multi-protein complexes in the nucleus or cytoplasm until ligand binding initiates their shuttling to chromatin DNA. To alter transcription, these receptors can bind directly to hormone response elements on DNA and/or interact with other transcriptional regulators [198, 199]. Notably, ERs and ARs can also be found as membrane receptors that have faster (non-classical) influences on neuronal function and/or transcriptional activity by modulating second messenger pathways and ion channels [200, 201]. Although androgen’s non-classical membrane actions were thought to largely occur through aromatization to E2, there is emerging evidence that T and/or its metabolites may act directly at membrane receptors. For example, 3α-diol has high affinity for GABA/benzodiazepine receptors [200], and actions at these receptors can decrease anxiety-like behavior (reviewed in [202]). In terms of the HPA axis literature, studies examining non-genomic gonadal steroid signaling are largely missing. However, there is evidence that E2 binds to a G-protein-coupled ER to desensitize serotonin signaling in the PVN and reduce activation of the HPA axis [203, 204].

## Role of neuropeptide systems in androgen regulation of stress

Studies spanning the last three decades have demonstrated a clear role for androgens in modulating neuropeptide systems that control the HPA axis and behavioral stress responses. In this section, we review androgen regulation of three key neuropeptide systems: CRF, arginine vasopressin (AVP), and oxytocin, and their cognate receptors.

### Corticotropin releasing factor

Corticotropin-releasing factor (CRF) signaling through pituitary CRFR1 is well established as critical for controlling the HPA axis [205]. CRF release and subsequent binding to brain cognate CRFR1 and CRFR2 receptors have also been repeatedly shown to regulate stress-associated behaviors as well as to modulate HPA axis tone [205, 206]. Genetic deletion or antagonism of CRFR1 suppresses the release of ACTH and CORT and decreases anxiety- and depressive-like behaviors [207–209], while CRFR1 stimulation produces opposite effects [210]. In contrast, genetic deletion of CRFR2 produces effects opposite to those of CRFR1 deletion, increasing anxiety-like behaviors and HPA responses to stress [211, 212]. These studies and others suggest opposing actions of CRFR1 and CRFR2 whereby CRFR1 binding increases activation of the HPA axis and anxiety-related behaviors while CRFR2 attenuates these responses.

Androgens have been demonstrated to produce brain region-specific alterations in CRF and both receptor phenotypes. Androgens are able to induce these effects due to the presence of androgen and estrogen response elements (AREs, EREs) in the promotor region of CRF and its cognate receptors [213–215]. Immunohistochemical studies also indicate that select groups of CRF and CRFR1 expressing neurons co-express androgen and estrogen receptors [27, 155, 171, 216]. For example, neurosecretory PVN CRF neurons are reported to show moderate co-expression of ERβ in both rats and mice [171, 188]. Additionally, while few CRF neurons co-express AR [27, 152], the majority of CRFR1-containing cells in the PVN co-express AR [155]. In the BST, a high percentage of CRF neurons also co-express AR [27]. Furthermore, CRFR2 and AR distributions show extensive overlap in the brain [217].

Several studies indicate that androgens can regulate CRF levels in the adult rodent brain. Bingaman et al. [218] demonstrated that CRF cell number in the PVN increased 3 weeks after GDX in males and treatment with DHT reversed this effect. Furthermore, restraint-induced elevations in PVN CRF hnRNA are reduced in GDX’d males supplemented with DHTP [180]. CRF mRNA levels are also reported to decrease in castrated male rats within the fusiform nucleus of the BST, and again, levels are restored following androgen supplementation [219]. An opposite effect of androgens on CRF occurs in the dorsolateral BST which contains a greater number of CRF neurons in female than male mice and rats [220, 221]. In the dorsolateral BST, castration decreases CRF levels in males [220]. Neonatal androgens have also been reported to alter adult levels of CRF. Specifically, treatment with the anti-androgen flutamide during the neonatal period increases CRF mRNA in the PVN of lipopolysaccharide-injected adult rats [222]. Furthermore, neonatal T treatment in female rats decreases the number of CRF-expressing cells within the dorsolateral BST in adulthood [223].

Sex differences have been reported in various brain regions for CRFR1 and CRFR2 (e.g., [155, 216, 224, 225]), and androgens have been demonstrated to play a role in regulating these sex differences in select brain areas. For example, CRFR1 levels are greater in the male compared to female PVN, and GDX in males decreases CRFR1 to levels similar to females [27, 155]. CRFR2 has also been shown to be upregulated by androgens in several brain regions. Treatment with DHTP increases CRFR2 mRNA levels within the lateral septum, hypothalamus, and hippocampus [226]. Altogether, these findings reveal that androgens affect the CRF system in a manner that is predicted to suppress the HPA axis response as well as anxiety- and depressive-like behaviors. For instance, high levels of PVN CRF are associated with hyper-activation of the HPA axis, whereas PVN CRFR1 has been shown to actively inhibit CRF neurons, thereby suppressing HPA axis function [153, 154]. Androgen-induced decreases in CRF and increases in CRFR1 would therefore be expected to diminish the HPA axis response. Furthermore, androgen-induced increases in CRFR2 might likely produce an anxiolytic effect given the known role of CRFR2 in reducing anxiety-related behaviors [211, 212].

### Arginine vasopressin

Arginine vasopressin (AVP) is produced in neurons in several brain regions including PVN, supraoptic nucleus, BST, and medial amygdala (MeA). Central release of AVP has been demonstrated to affect a wide range of behaviors including those associated with anxiety and depression [227]. AVP is also critical to HPA axis regulation; AVP released from the PVN acts in conjunction with CRF to potentiate the release of ACTH [228].

Several sex differences in the AVP system have been reported in rodents, including a greater number of cells expressing AVP in the male BST and MeA as well as a greater AVP fiber density in the lateral septum of males [229–231]. In contrast, AVP cell number in the PVN is commonly reported as similar between the two sexes [232], although within-sex studies have demonstrated that gonadal steroid hormones can regulate PVN AVP levels [150, 219, 233, 234]. Treatment of GDX’d males with E2 decreases PVN AVP mRNA in wild type, but not ERβ knockout mice, suggesting that this suppressive effect of estrogen is mediated through ERβ [233]. In rats, estrogens have been shown to regulate PVN AVP immunoreactivity in females [234]. Studies in male rodents also suggest a role of androgens in regulating PVN AVP. Implants of DHT into the posterior BST increase AVP mRNA in the PVN, while co-treatment with the anti-androgen, hyroxyflutamide, eliminates this effect [150]. Furthermore, GDX attenuates the rise in PVN AVP that occurs in response to adrenalectomy, while T or DHT replacement restores the elevated AVP response [219]. Since AR is largely absent in AVP neurons of the PVN, it has been proposed that androgens might act remotely in regions such as the BST to regulate AVP in the rat PVN [149, 150, 235]. However, AVP neurons in the PVN co-express ERβ indicating that androgen metabolites can directly act on these neurons [189, 236, 237].

Androgens potently regulate the sexually dimorphic distributions of AVP neurons and fibers. Long-term GDX in male rats (15 weeks) virtually eliminated cell bodies for AVP in the BST and MeA [238]. Accordingly, fiber density in the lateral septum was also diminished, since AVP fiber innervation to this area originates from the BST and MeA [229]. Subsequent studies indicate that T actions at both estrogen and ARs are necessary for masculinization of these structures. A series of studies performed by De Vries, Wang, and colleagues demonstrated that E2 increased AVP mRNA in the BST and MeA and a combined E2 + DHT treatment induced a further increase [239, 240]. AVP neurons in both regions highly co-express both AR and ERs indicating the presence of receptors through which E2 and DHT can produce these effects [235, 241]. Exogenous T treatment has also been shown to cause DNA hypomethylation of the AVP promoter in the MeA and BST [242] and in vitro studies identified ERβ binding to 3β-diol as a potential regulator of the AVP 5’untranslated region [243].

Fewer studies have investigated effects of androgens on AVP receptors (V1a, V1b, V2), and investigations in mammals have been primarily performed in hamsters with a focus on the V1a receptor (see [244] for review). Studies in Syrian hamsters indicate that castration decreases V1a receptor binding in the MPOA, ventromedial hypothalamus, and BST [245, 246]. Furthermore, androgens have been reported to increase V1a receptor mRNA in the medial preoptic area of Syrian hamsters [246]. However, studies in rats have failed to provide evidence for gonadal steroid hormone regulation of V1a [247, 248], indicating species differences in androgen sensitivity to effects on AVP and its receptors.

With regard to behavior, alterations in these AVP systems have primarily been linked to androgen-related modifications in social and aggressive behaviors [230, 244]. However, androgen-dependent changes in central AVP also have implications for modifying the HPA axis and stress-related behaviors. For instance, AVP release into the lateral septum has been shown to reduce immobility in rats in the forced swim test while infusion of a V1 antagonist into the same region produces opposite effects [249]. Since AVP innervation of the lateral septum is androgen regulated, this might be a mechanism through which androgens can modulate depressive-like behaviors.

### Oxytocin

Oxytocin has been repeatedly shown to reduce activation of the HPA axis and suppress anxiety-like behaviors [250–252]. Intracerebroventricular administration of oxytocin attenuates ACTH and CORT responses to stress, decreases anxiety-like behaviors, and decreases neural activation in the PVN [252–254]. Conversely, antagonism of the oxytocin receptor increases anxiety-like behaviors and HPA axis responses to stress [250, 255]. Oxytocin knockout models also reveal hyperactivation of the HPA axis after stress, further confirming the HPA axis-suppressing role of oxytocin signaling [256].

Oxytocin neurons in the PVN highly co-express ERβ but not ERα in the human and rodent PVN [171, 185, 188]. AR is also co-expressed within oxytocin neurons in the medial parvocellular division of the PVN (mpvPVN) but not in magnocellular oxytocin neurons in rats [235]. In humans, oxytocin/AR co-labeled neurons are also found in the PVN [257] and T treatment of a human neuroblastoma cell line reduces oxytocin mRNA through an AR-mediated mechanism [257]. Together these studies indicate the potential for androgens and their metabolites to regulate expression and function of PVN oxytocin neurons. E2 treatment increases oxytocin mRNA in the mouse PVN [233, 258]. Furthermore, these effects of E2 are dependent on ERβ since oxytocin mRNA levels are unaffected by E2 treatment in ERβ knockout mice [233, 258]. Since DHT can be converted to 3β-diol, which has high affinity for ERβ, this might be a pathway through which androgens can alter oxytocin expression. Evidence for this is reported by Sharma et al. [259] who utilized a mouse hypothalamic cell line which expresses both ERβ and oxytocin and found that treatment with the T metabolite 3β-diol, as well as DPN and E2, increased oxytocin mRNA levels. Further support for 3β-diol regulation of the oxytocin promoter comes from studies by Hiroi et al [260] who demonstrated that 3β-diol can activate the human oxytocin promoter through ERβ in a fashion that is consistent with the presence of a composite hormone response element in the 5’ untranslated region.

Gonadal steroid hormones have also been shown to induce changes in oxytocin receptor binding in rodents. For example, castrated rats treated with testosterone propionate (TP) show 4–5-fold increases in oxytocin binding in the BST and ventromedial hypothalamus [261]. E2 treatment also increases oxytocin receptor binding in the ventromedial hypothalamus and medial amygdala of rats and mice [258, 262]. Gonadal hormone regulation of oxytocin receptor in the ventromedial hypothalamus and medial amygdala appears to be primarily mediated by ERα, rather than ERβ, since E2 increases oxytocin receptor binding in ERβ knockout mice [258]. Together, these studies provide evidence that androgens can potentially mediate levels of oxytocin and receptor binding following metabolism to estrogenic compounds. Additionally, an oxytocin antagonist can block some of the effects of the ERβ agonist DPN on anxiety-like behaviors [263]. Thus, the HPA axis-suppressing and anxiety-reducing effects of oxytocin might be modulated through these mechanisms.

## Perspectives and significance

As described in this review, androgens play a key role in influencing the HPA axis and anxiety- and depressive-like behaviors and, accordingly, contribute to sex differences in these functions. Although substantial work has been done to determine the androgen metabolites and receptor subtypes involved in modulating the HPA axis and behavioral stress responses [3], questions remain regarding critical periods during which androgens act to produce these effects. Another key question remains with regard to the specific cell phenotypes and anatomical regions involved in androgen effects on these responses. Important to consider are the potentially opposing hormonal and behavior effects induced by androgen and androgen metabolites acting within different areas. Determining the precise sites of action that regulate these functions is difficult given the widespread distribution of gonadal steroid hormone receptors (e.g., AR, ERα, and ERβ) in numerous brain regions known to regulate stress responses [147, 172, 186]. Regardless, several brain regions have thus far been indicated as key central sites of actions including the PVN, peri-PVN, BST, dorsal raphe, hippocampus, and nucleus accumbens [3, 70, 174]. Furthermore, key cell phenotypes including those that produce or contain receptors for CRF, AVP, and oxytocin appear critical for the influence of androgens on the HPA axis and stress-related behaviors. Future studies that utilize contemporary viral and genetic strategies will be key in elucidating the precise mechanisms through which androgens regulate stress responses. Uncovering these mechanisms will thus provide essential insight toward understanding how sex hormones contribute to sex disparities stress-related mental illnesses including anxiety and depression.

## Data Availability

Data scoring is not applicable to this article as no datasets were generated or analyzed during the current study.

## Abbreviations

3α-Diol: 5alpha androstane 3α,17β diol
3β-Diol: 5alpha androstane 3β,17β diol
17βHSD: 17β Hydroxysteroid dehydrogenase
ACTH: Adrenocorticotrophic hormone
ANS: Autonomic nervous system
AR: Androgen receptor
ARE: Androgen response element
AVP: Arginine vasopressin
BST: Bed nucleus of the stria terminalis
CBG: Corticosteroid-binding globulin
CORT: Corticosterone
Cre: Cre recombinase
CRH: Corticotropin-releasing hormone
CRFR1: Corticotropin-releasing factor receptor 1
CVS: Chronic variable stress
CYP7B: Cytochrome p450 7b
DHT: Dihydrotestosterone
DHTP: Dihydrotestosterone propionate
DPN: Diarylprionate
E2: Estradiol
ER: Estrogen receptor
ERE: Estrogen response element
ESR2: Estrogen receptor 2
GABA: Gamma aminobutyric acid
GC: Glucocorticoid
GDX: Gonadectomy
GR: Glucocorticoid receptor
HPA: Hypothalamic-pituitary-adrenal
iTFM: Induced testicular feminization mutation
MeA: Medial amygdala
MPOA: Medial preoptic area
MR: Mineralocorticoid receptor
POMC: Proopiomelanocortin
PPT: Propylpyrazoltriol
PVN: Paraventricular nucleus
RL-HSD: 11-cis-retinol dehydrogenase
T: Testosterone
Tfm: Testicular feminized mouse
TPH: Tryptophan hydroxylase
V1A: Vasopressin receptor 1A
V2A: Vasopressin receptor 2A
